# A multi-attention mechanism-improved ResNet18 approach for lithology identification and classification

**DOI:** 10.1371/journal.pone.0361625

**Published:** 2026-10-27

**Authors:** Yan Zhang, Fan Zhang, Jiaming Li, Lantao Huang, Ruifeng Li

**Affiliations:** 1 Guangxi Key Laboratory of Geomechanics and Geotechnical Engineering, Guilin University of Technology, Guilin, China; 2 Guangxi Key Laboratory of Green Building Materials and Construction Industrialization, Guilin University of Technology, Guilin, China; China University of Mining and Technology, CHINA

## Abstract

Lithology identification is vital for improving geological exploration accuracy, optimizing petroleum exploration schemes, and ensuring construction engineering safety. Traditional identification methods suffer from low efficiency, high cost, and heavy dependence on domain expertise. This study proposes a lithology identification approach based on the ResNet18 backbone, in which five lightweight attention mechanisms, namely CBAM, channel-only attention, ECA, SE, and spatial-only attention, are systematically compared, and the best-performing CBAM module is embedded to strengthen feature extraction and discrimination capacity. The Open-Source Computer Vision (OpenCV) library is adopted for dataset preprocessing, where raw images are converted to grayscale to suppress interference from irrelevant information. Comparative experiments demonstrate that CBAM outperforms the other evaluated attention modules and is therefore incorporated into the final network architecture. A hyperparameter sensitivity analysis of the CBAM-based model demonstrates that the model performance varies with different combinations of learning rate and batch size. When trained with a learning rate of 0.0001 and a batch size of 16, the model achieves a lithology identification accuracy of 86.36%, substantially improving overall recognition performance. By integrating CBAM into ResNet18, the proposed method enables accurate recognition to support multi-field engineering applications, reduce risks, and promote intelligent precision.

## 1. Introduction

Lithology identification and classification is a core technology in geological science that underpins the entire lifecycle of resource exploration and engineering construction. As emphasized by Han et al. [1], accurate lithology identification not only provides critical insights for regional tectonic analysis but also directly determines the efficacy of mineral resource prospecting [2]. In petroleum exploration and development, precise lithological analysis yields reservoir lithological characteristics that enable accurate assessment of hydrocarbon productivity potential and development value [3,4]. In underground construction scenarios such as tunnel engineering, the significant disparities in physical and chemical properties across different lithologies directly induce variations in engineering behavior, which in turn affect construction safety and schedule management. Relevant mechanical studies have further demonstrated that environmental factors including high temperature and water content can significantly change the fracture characteristics and rockburst proneness of granite, further increasing the complexity of engineering risk control. [5,6] Accurate lithology identification is therefore an indispensable part of engineering risk assessment and construction planning [7,8]. Furthermore, research by Yan and Song [9] has shown that lithology identification technology offers unique advantages in evaluating building foundation stability and selecting engineering materials, underscoring its foundational role in geological research and engineering practice.

Traditional rock identification methods, such as on-site observation, thin section analysis, and chemical characterization, ensure high reliability. However, their low efficiency, high cost, and dependence on experts limit their adaptability to the digital transformation of exploration technology [10]. In contrast, computer vision and deep learning have driven intelligent lithology identification based on image analysis, with deep Convolutional Neural Networks (CNNs) performing well in classifying rock texture, color, and structural features [11,12]. Recent studies have proposed various methods in this direction. Liu et al. [13] combined deep learning with K-means for rock mineral identification; Senjoba et al. [14] integrated drill bit vibration signals with deep learning for automation; Gao et al. [15] developed a cost-effective method for identifying high-temperature rocks from Scanning Electron Microscope (SEM) images; A study applied object detection technology to realize intelligent identification of shale pores and minerals, providing a practical solution for refined shale reservoir evaluation [16]. An improved U-Net deep learning model was further proposed to achieve fine-grained pore characterization based on SEM images, effectively improving the extraction accuracy of pore structure parameters [17]. Moreover, a specialized Pore-Net network was developed for intelligent identification and quantitative characterization of pores in shale SEM images, which further enriches the technical system of digital petrographic analysis [18]. Zhang et al. [19] introduced a time-frequency spectrogram-based deep learning method for determining rock joint roughness, which achieves higher accuracy in rock classification; Xu et al. [20] compared Inception-v3 and other models for identifying rock properties; Li et al. [21] used CNNs to predict the intensity grade, occurrence time, and location of rockbursts; to improve the reliability of monitoring data, an adaptive conditional denoising network (CDU-Net) was developed for microseismic signals in complex underground environments, which provides solid data support for rockburst risk early warning [22]. Yan and Xiao [23] combined laser spectroscopy with deep learning for coal gangue classification; Zhao et al. [24] proposed a rock identification method based on Tunnel Boring Machine (TBM) vibration; and Luo et al. [25] applied a DeepLabV3 + semantic segmentation workflow to identify rock categories from images acquired via Unmanned Aerial Vehicle (UAV) oblique photography. Additionally, machine learning has been widely used for petrographic and lithology classification based on borehole geophysical logs and field data. Overall, most researchers focus on improving feature extraction quality by integrating other techniques or algorithms to enhance model accuracy and efficiency. However, such methods often involve complex processes, such as cross-technical parameter standardization, multi-algorithm interface adaptation, and multi-device collaborative training. These complexities make it difficult to meet the requirements under resource-constrained conditions, thus posing significant challenges.

Advanced deep convolutional neural network architectures effectively enhance feature extraction while maintaining implementation simplicity. Compared with deeper ResNet variants (e.g., ResNet50/101) and custom complex networks, ResNet18 is a relatively compact standard variant of the ResNet family, a milestone in deep learning that overcomes deep network training challenges via residual connections. Specifically, ResNet18 has only approximately 11.7M parameters and 1.8G MACs, substantially fewer than ResNet50 (25.6M parameters, 4.1G MACs). Its compact architecture, combined with a mature open-source ecosystem (e.g., official TorchVision provides one-line access to ImageNet-pretrained weights), results in a low deployment threshold and ease of implementation under resource-constrained conditions. Several studies have leveraged ResNet18 for different tasks and have attempted various modifications to further enhance its performance. For instance, Wang et al. [26] integrated Variational Mode Decomposition and Discrete Wavelet Transform (VMD-DWT) denoising with an improved ResNet18 to enhance feature capture, while Shan et al. [27] incorporated softpool and Efficient Channel Attention (ECA) into their ES-ResNet18 model to reallocate weights to critical feature maps. Other works have explored model fusion, such as Naz et al. [28] combining XcepNet23 with ResNet18, and Ullah et al. [29] integrating ResNet18 with Faster RCNN for object detection. Additionally, Li and Duan [30] modified ResNet18 for hyperspectral image analysis, and Xiao et al. [31] confirmed ResNet18’s superiority in self-compacting concrete quality recognition.

Although these studies successfully improved feature extraction quality and model accuracy, they also introduced notable limitations that motivate the present research. First, integrating ResNet18 with complex auxiliary algorithms (e.g., VMD-DWT, Faster RCNN) or multi-model fusion significantly increases network complexity, computational cost, and memory consumption, which hinders deployment in resource-constrained field environments. Second, although attention mechanisms (e.g., ECA) improve general feature representation, existing improved ResNet18 models often lack a targeted focus on the unique visual characteristics of rocks, such as texture, grain arrangement, and weathering color cast, resulting in limited accuracy under complex lithological scenarios. Therefore, there remains a clear need for an effective improvement to ResNet18 that can specifically enhance the extraction of intrinsic lithological features without excessive resource demands.

This study proposes an improved ResNet18-based method that integrates the CBAM attention mechanism to enhance lithology identification and classification performance. To address the aforementioned issues, the proposed method incorporates three key architectural modifications. First, the original average pooling layer is replaced with an adaptive average pooling layer to improve adaptability to varying input features. Second, the fully connected layer is renewed to enhance generalization and prevent overfitting, particularly given the limited scale of the dataset. Third, a single CBAM attention module is integrated into the network. Crucially, to maintain architectural simplicity and computational efficiency, this module is inserted after the entire convolutional backbone (i.e., after the last BasicBlock of Layer4) and immediately before the adaptive average pooling layer. This placement allows the model to perform a global recalibration of deep features without altering the internal structure of individual BasicBlocks. Together, these improvements optimize feature extraction, suppress irrelevant interference, and provide a robust and practical solution for lithology identification tasks.

The main methodological innovation of this work lies in a systematic empirical study and a simple integration scheme, rather than in proposing a novel attention module per se. Specifically, our contributions are threefold. (1) Comprehensive benchmarking: we present, for the first time, an end-to-end performance comparison of five prevalent attention mechanisms (CBAM, Channel, SE, ECA, and Spatial) within the ResNet18 architecture, specifically tailored to rock image classification with a small-scale dataset. (2) In-depth analysis and validation: beyond identifying the best-performing mechanism (CBAM), we provide an in-depth analysis through multi-metric evaluation, confusion matrices, and hyperparameter sensitivity analysis, offering a validated and reproducible solution. (3) Practical, plug-and-play solution: the proposed improvement, integrating a single attention module after the ResNet18 backbone while employing adaptive pooling, avoids complex architectural changes and yields an effective model for practical lithology identification.

## 2. Model fundamentals and construction

### 2.1 ResNet18 network architecture

As a classic residual network proposed by He et al. [32], ResNet18 introduces shortcut connections between convolutional layers, which effectively alleviates the gradient vanishing problem and network degradation caused by increasing network depth. Through identity mapping of residual branches, the deep network maintains stable feature transmission during training, ensuring that the model can still effectively learn hierarchical features as the number of layers increases. The network structure of ResNet18 is illustrated in Fig 1.

**Fig 1:**
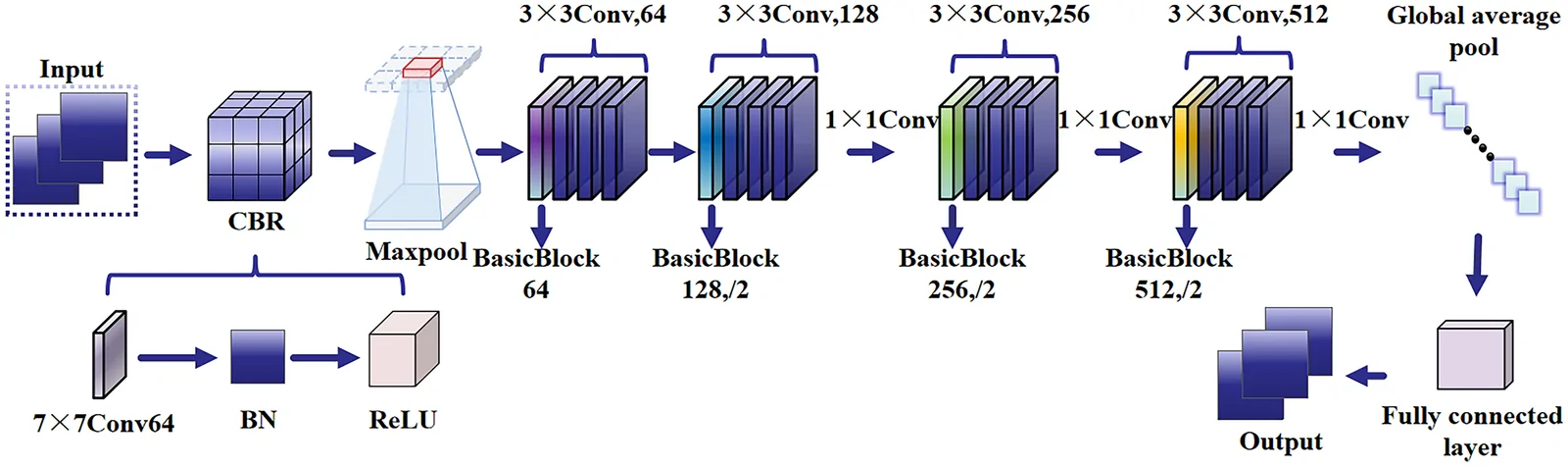
Network diagram of the ResNet18 model.

The ResNet18 architecture consists of five parts. First, a 7 × 7 convolution layer with batch normalization (which normalizes feature distributions to stabilize training) is applied, followed by a ReLU activation function (which introduces non-linearity to enable learning of complex features) and a 3 × 3 max pooling layer (which reduces the spatial dimension to improve efficiency). This initial module extracts low-level visual features.

After that, four stacked residual stages are included, each composed of multiple BasicBlock units. Each BasicBlock contains two 3 × 3 convolutional layers and a shortcut connection. Finally, a global average pooling layer compresses features to one dimension, and a fully connected layer outputs classification probabilities.

### 2.2 Fundamentals of attention mechanisms

However, traditional CNN models assign equal weights to all regions, which may result in information loss and compromised accuracy. To address this issue, various attention mechanisms have been proposed to enable networks to focus on key features, such as the Convolutional Block Attention Module (CBAM).

CBAM [33] is an attention module for CNNs that adaptively focuses on different regions of an image to improve model performance. It consists of a channel attention module and a spatial attention module. The channel attention module generates channel feature vectors by applying global average and max pooling on the feature map. These vectors are then processed through a shared Multi-Layer Perceptron (MLP) and normalized using a sigmoid function to obtain channel attention weights. Finally, these weights are multiplied by the original feature map to produce the channel attention-enhanced feature map.

The resulting feature map is then fed into the spatial attention module, which applies average and max pooling across the channels to obtain spatial feature maps. These maps are concatenated and processed by a convolutional layer to generate spatial attention weights. Finally, the spatial weights are multiplied by the feature map to obtain the spatial attention-enhanced feature map.

Mathematical formulation of CBAM: Given an intermediate feature map $𝐅\in {\mathbb{R}}^{C\times H\times W}$ as input, the channel attention module first applies global average and max pooling along the spatial dimensions to obtain channel-wise descriptors, which are then passed through a shared multi-layer perceptron. The channel attention weight $M_{c}$ is computed as Eq. 1:


$$\begin{array}{c} {𝐌}_{c}\left(𝐅\right)=\sigma \left(\text{MLP}\left(\text{AvgPool}\left(𝐅\right))+\text{MLP}(\text{MaxPool}\left(𝐅\right)\right)\right) \end{array}$$ (1)

where σ denotes the sigmoid activation. The resulting feature map ${𝐅}^{\prime }={𝐌}_{c}\left(𝐅\right)⊗𝐅$ is then fed to the spatial attention module, which applies channel-wise pooling followed by a 7×7 convolution Eq. 2:


$$\begin{array}{c} {𝐌}_{s}\left({𝐅}^{\prime }\right)=\sigma \left(f^{7\times 7}\left(\left[\text{AvgPool}\left({𝐅}^{\prime });\text{MaxPool}({𝐅}^{\prime }\right)\right]\right)\right) \end{array}$$ (2)

The final recalibrated output is ${𝐅}^{\prime \prime }={𝐌}_{s}\left({𝐅}^{\prime }\right)⊗{𝐅}^{\prime }$. This sequential channel-spatial interaction enables the model to first select informative feature channels and then focus on salient spatial regions, which is particularly beneficial for capturing rock texture patterns [34].

The structure of the CBAM attention module is demonstrated in Fig 2.

**Fig 2:**
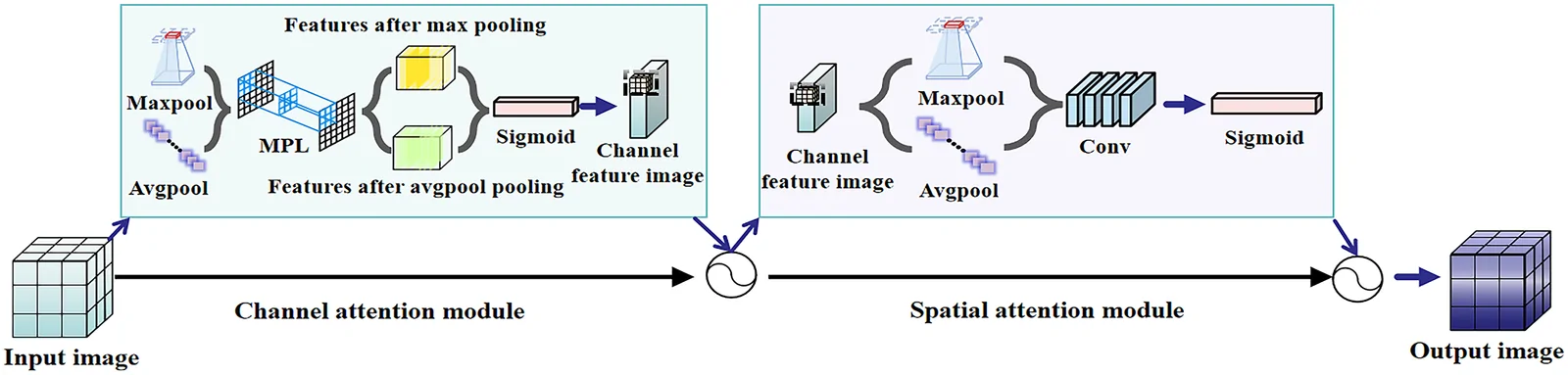
Structure of the CBAM.

Channel attention [33] is an efficient feature screening and enhancement strategy that optimizes information processing capability in terms of feature types. In the channel dimension of the feature map, key channels are selected by learning weights, while irrelevant information is suppressed. The core principle is as follows. First, in the global information aggregation stage, the feature map is compressed into channel vectors via global pooling to obtain the global representation of each channel. Second, in the channel weighting stage, importance weights are computed based on the channel representations, and non-linear transformations capture the dependencies between channels. Finally, in the feature weighting stage, the learned weights are multiplied by the original feature map to strengthen key channel information.

The spatial attention module [33], proposed by Woo et al., is a key technique in deep learning for enhancing the spatial information of feature maps. It explicitly performs spatial transformation on feature maps, enabling the model to focus on key regions while suppressing irrelevant information. Its core principle is as follows. First, average pooling and maximum pooling are performed along the channel dimension, respectively, to obtain spatial feature descriptors. Then, the descriptors are compressed to a single channel through a convolution layer, and a spatial attention map is generated via the sigmoid function. Finally, the attention map is multiplied with the original features to strengthen the key spatial region features. The structural composition of the spatial attention module is outlined in Fig 2.

The Squeeze-and-Excitation (SE) module [35], proposed by Hu et al., is a classic channel attention mechanism in deep learning. It dynamically adjusts the contribution of each channel in the feature map by learning the importance weights of different channels, enabling the model to focus on key information while suppressing irrelevant information. Its core principle is as follows. First, the feature map undergoes global average pooling to obtain the global features of the channel dimension. Second, dimensionality reduction and restoration are performed through a two-layer fully connected network, and channel weights are generated using the ReLU and sigmoid functions. Finally, the weights are multiplied by the original feature map to obtain the reweighted features. The structure of the SE attention module is illustrated in Fig 3.

**Fig 3:**
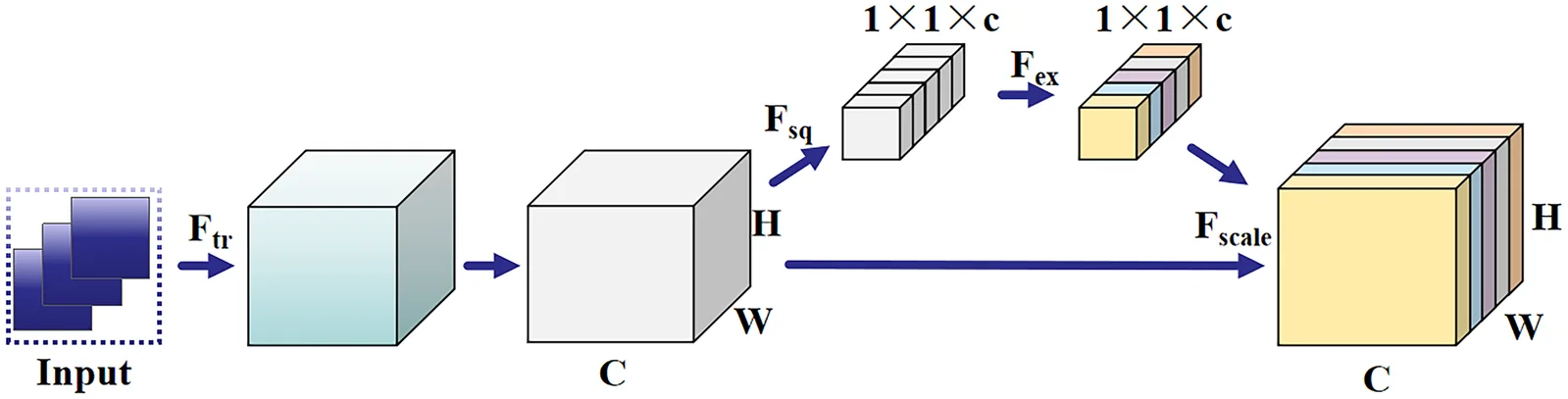
Structure of the SE.

The Efficient Channel Attention (ECA) module [36], proposed by Wang et al., is a lightweight channel attention mechanism that effectively addresses the problem of channel information loss caused by dimensionality reduction in traditional channel attention. Its core principle is as follows. First, channel features are extracted via global average pooling. Then, a one-dimensional convolution is applied along the channel dimension, where the kernel size is adaptively determined by a formula, achieving local cross-channel interaction. After activation by the sigmoid function, the channel weights are multiplied by the original features to complete feature-weighted enhancement. The structure of the ECA attention module is shown in Fig 4.

**Fig 4:**
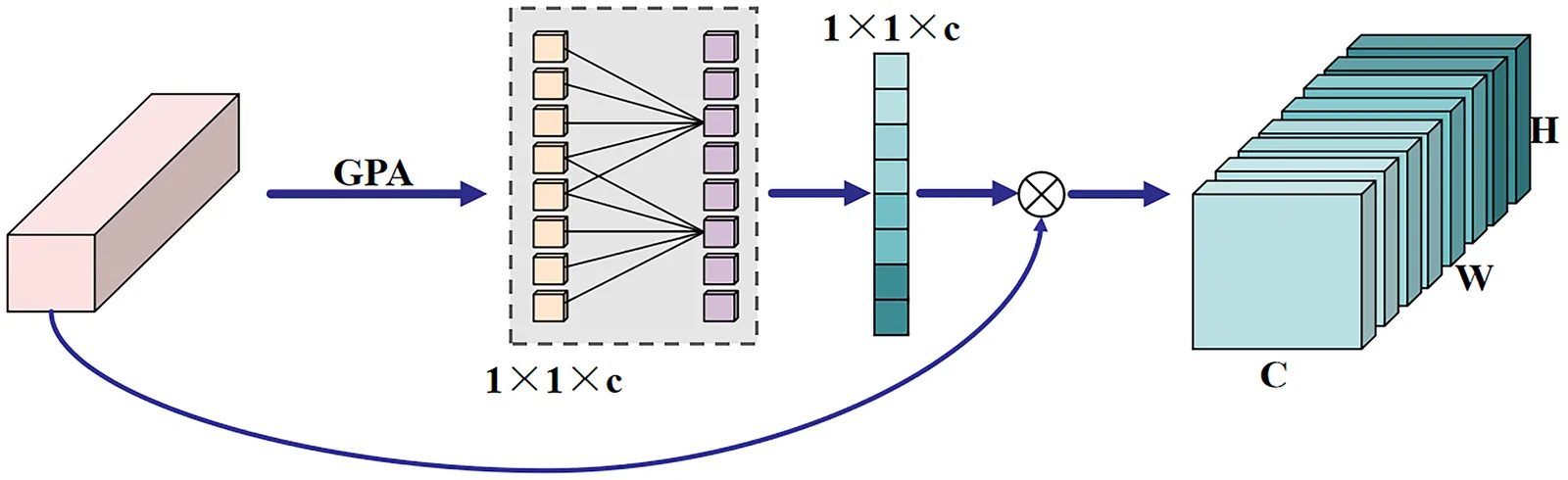
Structure of the ECA.

## 3. ResNet18 improvement method

The original ResNet18 model was designed to address the feature loss that occurs as network depth increases. Its unique layer-hopping connectivity maintains feature transfer as the network depth increases to 18 layers. However, the many layers and parameters of the residual structure mean that the training process consumes excessive memory and time. To overcome these issues, this study proposes an improvement to the ResNet18 model based on multiple attention mechanisms.

First, the average pooling and fully connected layers are removed from the original model. The average pooling layer compresses the feature map to a fixed size (7 × 7), which is designed to facilitate the subsequent processing of the fully connected layer, while the fully connected layer maps features to a fixed number of output categories. These two layers are primarily designed for task-specific pre-training: they can only handle the same number of categories as during training and cannot be adapted to new tasks. Therefore, removing these two layers improves the model’s flexibility to adapt to different task requirements.

Second, the generic features are retained and the feature task layer is replaced. The model’s convolutional layer extracts generic features of the image that are useful for training the model. Retaining the feature extraction part of the convolutional layer enables the model to learn generic features from the dataset more quickly, allowing it to achieve better results on new datasets faster.

Third, an adaptive average pooling layer is added. This unifies the output feature size, making it easier for the fully connected layer to compute a unified classification. Compared with the traditional average pooling layer, the adaptive average pooling layer can dynamically adjust the pooling window size and stride according to the size of the input feature map. This reduces the network’s parameter count and computational complexity and improves model efficiency.

Fourth, the fully connected layer is replaced. The primary purpose of this layer is to enable the model to improve its generalization ability, adapt to different task categories, and prevent overfitting, which is easily produced when the amount of data is limited. Replacing the fully connected layer allows the model structure to be adjusted flexibly, and it is convenient to tune the number of layers in the fully connected layer during the experimental process. This enables the model to find the network structure that produces the best classification results.

From an implementation perspective, the aforementioned replacements are applied without drastic modifications to the original architecture, so that the improved model retains the well-learned generic features of the pretrained backbone while adapting its task-specific layers to the lithology classification objective. Furthermore, the strategic placement of the attention module after the backbone, rather than within each residual block, simplifies the overall architecture and ensures that the model autonomously focuses on the most discriminative lithological features output by the entire network.

This design strategy avoids drastic modifications to the original network architecture and facilitates targeted feature optimization by incorporating the CBAM attention module. Drawing on the residual attention mechanism used for effective feature retention and noise suppression in microseismic signal processing [37], the attention module is added before the residual connection to retain critical lithological features and suppress irrelevant interference. In addition, referring to the adaptive modeling idea for dynamic signal characteristics [38], an adaptive average pooling layer is adopted to replace the traditional average pooling layer, which enhances the model’s adaptability to diverse lithology image features. This mechanism moderates the incremental increase in complexity while improving the feature extraction capability of the model, enabling it to concentrate on high-value feature regions. The resulting feature representations are more discriminative for subsequent classification tasks, enhancing the performance of the ResNet18 model. The network architecture of the proposed model is shown in Fig 5.

**Fig 5:**
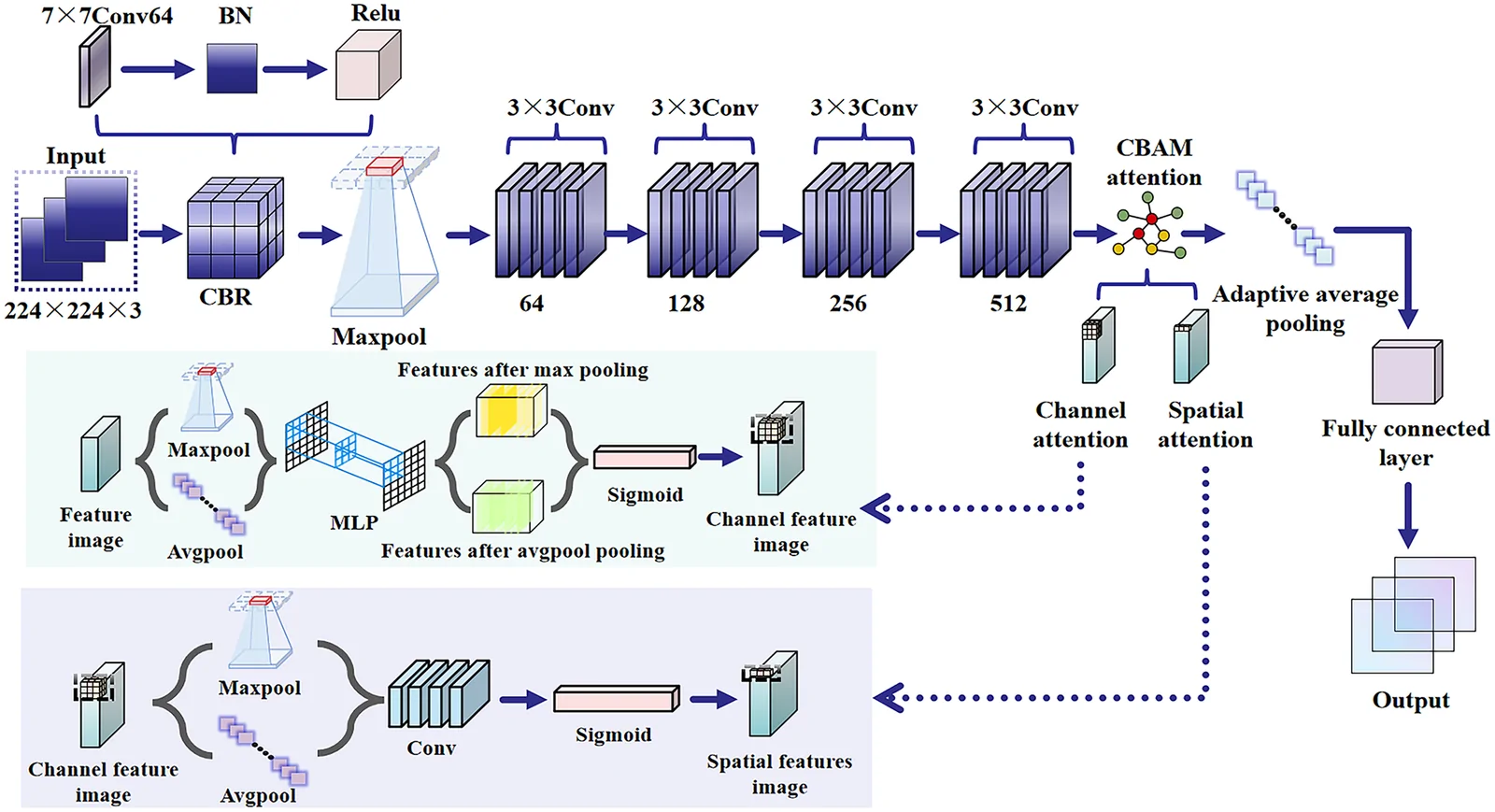
Architecture of the proposed CBAM-ResNet18 model. The CBAM attention module is inserted after the fourth convolutional block (Layer4) and before the adaptive average pooling layer. The diagram details the internal structure of the CBAM module, which consists of a channel attention branch (top) and a spatial attention branch (bottom) operating sequentially.

## 4. Comparison of multiple methods and analysis of results

### 4.1 Assessment of indicators

All experiments were conducted on the same hardware platform: an Intel Core i7-12700H CPU, an NVIDIA GeForce RTX 3060 GPU (6GB VRAM), and 16GB RAM. The software environment includes Python 3.8, PyTorch 1.12.1, OpenCV 4.5.5, and CUDA 11.3.

To rigorously evaluate the performance of different methods, this study employs multiple evaluation metrics: accuracy (Acc), precision (P), recall (R), loss value (Loss), and F_1_-score. These metrics collectively form a comprehensive framework for verifying the model’s identification accuracy.

Accuracy (Acc), a fundamental metric for assessing classification performance, is mathematically defined in Eq. (3):


$$\begin{array}{c} A\text{cc =\textasciitilde{}}\frac{TP+TN}{TP+TN+FP+FN} \end{array}$$ (3)

where *TP* denotes the number of positive samples correctly identified by the model; *TN* represents the count of negative samples correctly classified; *FP* refers to negative samples incorrectly predicted as positive, and *FN* is the count of positive samples mistakenly classified as negative.

Precision (*P*), a key metric for evaluating the reliability of positive predictions, is formally defined in Eq. (4):


$$\begin{array}{c} P=\frac{TP}{TP+FP} \end{array}$$ (4)

Recall (*R*), also referred to as sensitivity, quantifies the proportion of actual positive samples correctly identified by the model, as formally expressed in Eq. (5):


$$\begin{array}{c} R=\frac{TP}{TP+FN} \end{array}$$ (5)

Loss value (*Loss*), which quantifies the deviation between the model’s predicted results and the true labels, is typically calculated via a loss function, as formally defined in Eq. (6):


$$\begin{array}{c} Loss=-\frac{\text{1}}{n}\sum_{i=1}^{n}\sum_{j=1}^{m}y_{ij}log\left(y_{ij}^{\prime }\right) \end{array}$$ (6)

where $y_{ij}$ is the binary true label of the *i*-th sample for the *j*-th category y^ij is the model-predicted probability that the *i*-th sample belongs to the *j*-th category. The *F*_1_-score, a balanced metric that synthesizes precision and recall, is formally defined as the harmonic mean of *P* and *R* in Eq. (7):


$$\begin{array}{c} F_{\text{1}}-score=2\times \frac{P\times R}{P+R} \end{array}$$ (7)

where P and R are the precision and recall values defined earlier, ensuring a comprehensive assessment that avoids bias toward either metric. These metrics encapsulate essential information about the model’s predictive performance and serve as critical indicators for analyzing its efficacy in lithological identification.

### 4.2 Dataset construction and preprocessing

Machine vision methods can extract texture, contour, and structural features from rock surface images, which serve as the primary visual basis for lithology differentiation. In this study, grayscale preprocessing is adopted to mitigate the interference of uneven illumination and environmental color cast, enabling the model to focus on the intrinsic texture and structural characteristics of rocks. The rock dataset used in this study is compiled from publicly available images in open-source scientific databases, namely Salman [39] and Rem-ml8k [40], by extracting and merging relevant samples from both sources. It comprises 1,093 images covering seven rock types: basalt, coal, limestone, calcitic marble, rhyolitic pumice, quartzite, and rhyolite. The dataset was randomly split into training and test sets at an 8:2 ratio. The seven lithologies and their key visual and geological characteristics are summarized as follows: basalt exhibits a compact massive structure with fine vesicles and a uniform texture; coal typically presents a black, layered or banded structure with a dull to vitreous luster and a relatively soft, fine-grained matrix; limestone displays distinct bedding structures and a noticeable granular fabric; calcitic marble shows directional banded textures formed by metamorphic recrystallization; rhyolitic pumice is characterized by a highly vesicular, frothy texture with visible flow banding; quartzite is distinguished by its high hardness and smooth, splintery fracture surface; and rhyolite presents a fine-grained to aphanitic texture with distinct flow structures and a uniform felsic appearance.

During preprocessing, all original images are first converted to single-channel grayscale using OpenCV functions in Python, followed by noise reduction and histogram equalization for image enhancement. The resulting grayscale images are then replicated into a three-channel format to match the input dimension requirements of the ResNet18 backbone. This grayscale strategy filters out interference from uneven illumination, surface weathering, and environmental humidity, thereby avoiding the instability of color features commonly encountered in field rock image acquisition. Although this step inevitably discards color information that could potentially help distinguish mineralogically similar lithologies, it substantially reduces computational complexity and mitigates the risk of overfitting to color variations caused by inconsistent lighting conditions across different acquisitions. The primary discriminative features for lithology identification, namely texture, grain arrangement, and surface morphology, are preserved in grayscale. We acknowledge that multispectral or color-based feature extraction [41] may provide complementary information, and we leave this as a direction for future work. Upon completion of this pipeline, the refined dataset is obtained and prepared for subsequent model training and evaluation. The detailed implementation steps are illustrated in Fig 6.

**Fig 6:**
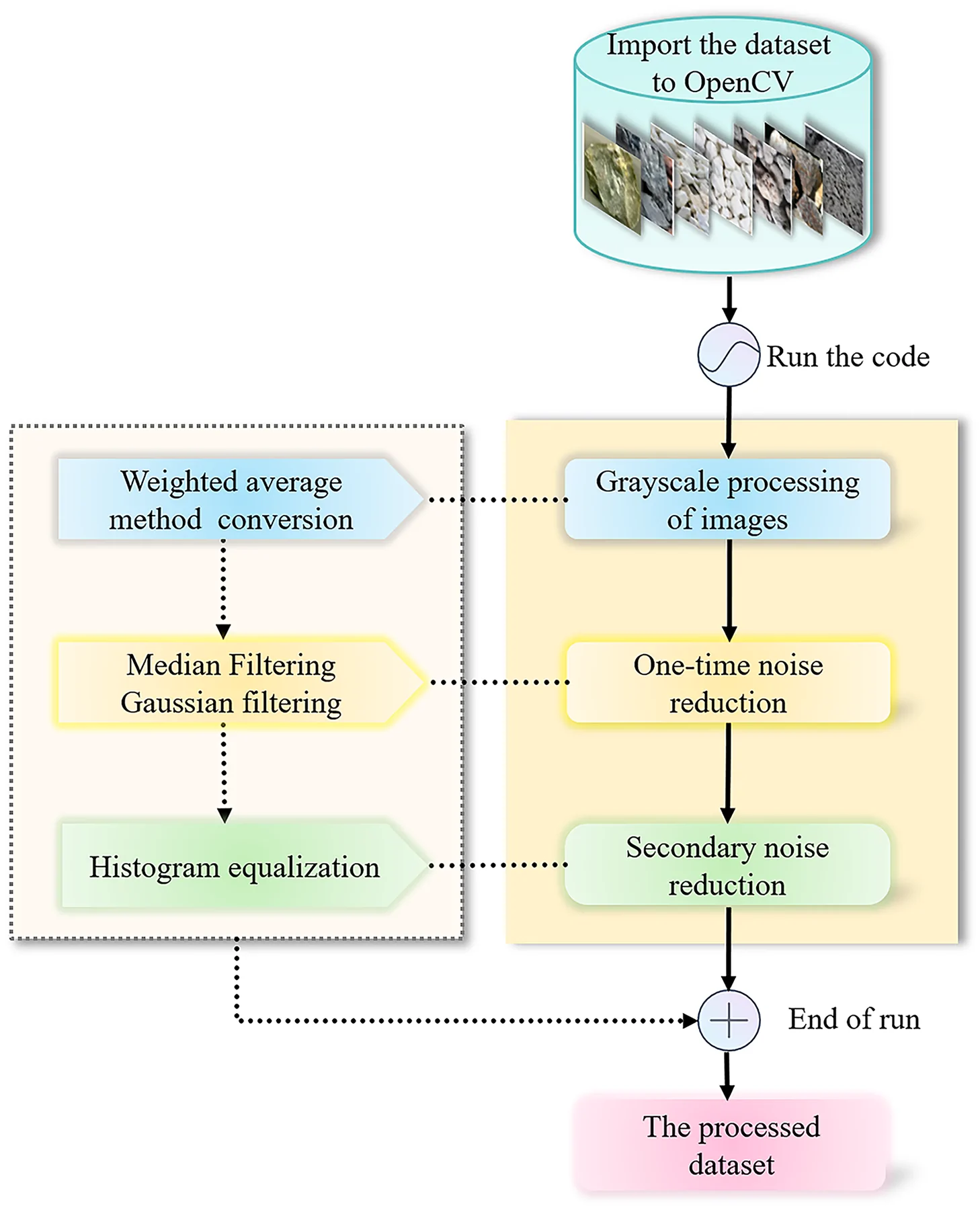
OpenCV image processing flowchart.

After grayscale processing, the interference of color information in rock images can be eliminated, which helps the model focus on morphological and textural structural features and provides a unified visual basis for rock category identification and classification. Specifically, the color image is converted into a grayscale image that retains only brightness information. This process converts the RGB (red, green, and blue) pixels of the three-channel image into a single grayscale value, merging the three channels of the original color image into one. The data volume is reduced to one-third of the original, which is conducive to improving the efficiency of model training and inference. The principle of this conversion method is depicted in Eq. (8).


$$\begin{array}{c} Gray=0.299R+0.587G+0.114B \end{array}$$ (8)

Noise reduction is a key step in the image preprocessing pipeline. It suppresses various noise sources introduced during image acquisition and transmission and ensures the integrity of effective image features. This process helps improve visual clarity, which is particularly important when noise may obscure the identification of rock textures and structural features. Effective noise reduction can refine the feature presentation of the image, thereby reducing the risk of feature misjudgment by the model.

Image enhancement processing adjusts the distribution of gray values in the image, amplifies the difference between different gray levels, and improves the overall contrast of the image. This process can highlight the texture contours and other salient structural features of rocks and weaken the interference of irrelevant background information, so as to assist the model in extracting more discriminative features. After conversion to single-channel grayscale images, all samples are replicated three times along the channel dimension to generate three-channel images with a size of 224 × 224 × 3. This operation matches the input dimension requirement of the ResNet18 backbone while retaining only grayscale texture information without introducing additional color features (Fig 7).

**Fig 7:**
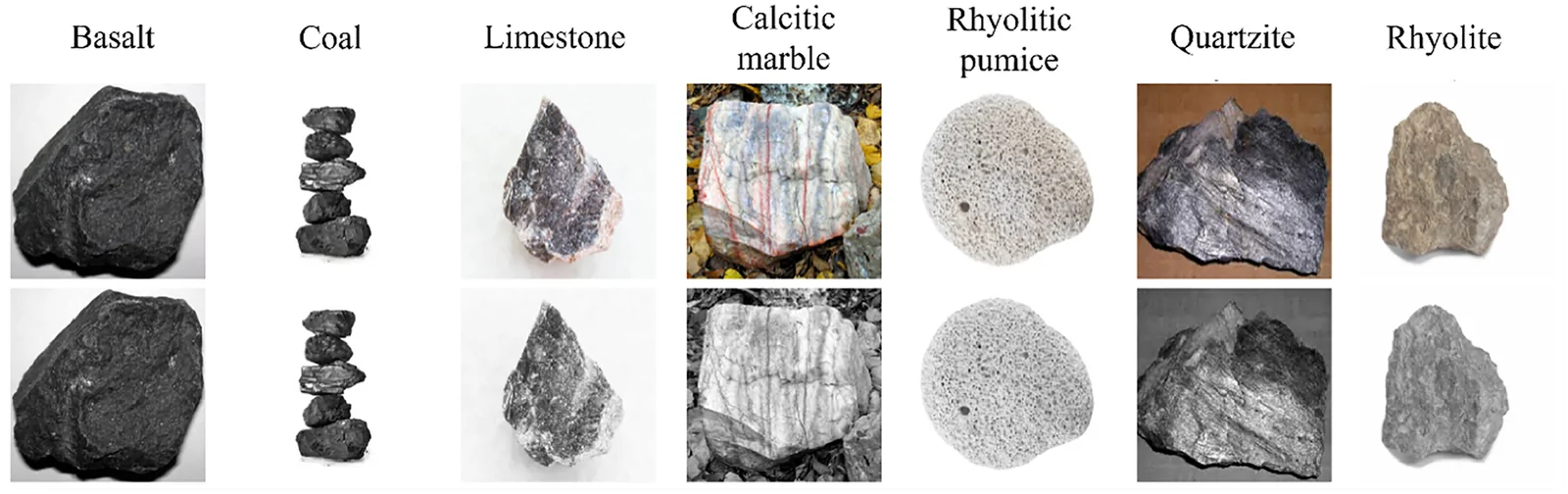
Visual comparison of original RGB images and grayscale preprocessed images for representative rock samples.

We trained the identical ResNet18-CBAM architecture under a consistent training protocol and 5-fold cross-validation scheme for two input formats, respectively: (1) original RGB color images; and (2) single-channel grayscale images replicated to three channels along the channel dimension to match the input dimension of the model. The quantitative results show that the grayscale-input model achieves a test accuracy of 86.36%, slightly outperforming the RGB-input model at 85.69%. The same trend is observed across the precision, recall, and F_1_-score metrics. This result indicates that color information does not provide additional discriminative value on this dataset but instead interferes with model performance.

The underlying reason lies in the heterogeneity of imaging conditions across the merged open-source datasets. As can be seen from the sample images, the surface color of rocks even within the same lithology varies significantly due to factors such as inconsistent ambient lighting, varying degrees of surface weathering, differences in moisture content, and different camera white balance settings during acquisition. These unstable chromatic variations constitute dataset-specific noise rather than inherent lithological features of rocks, which increases the risk of the model overfitting to spurious color cues. In contrast, grayscale preprocessing eliminates such chromatic interference and drives the model to focus on texture, grain arrangement, and structural features.

Following image preprocessing, the final dataset comprises 156 basalt images, 153 coal images, 148 limestone images, 174 calcitic marble images, 136 rhyolitic pumice images, 163 quartzite images, and 163 rhyolite images. The training and test sets account for 80% and 20% of the data, respectively. The specific sample distribution of the dataset is shown in Fig 8.

**Fig 8:**
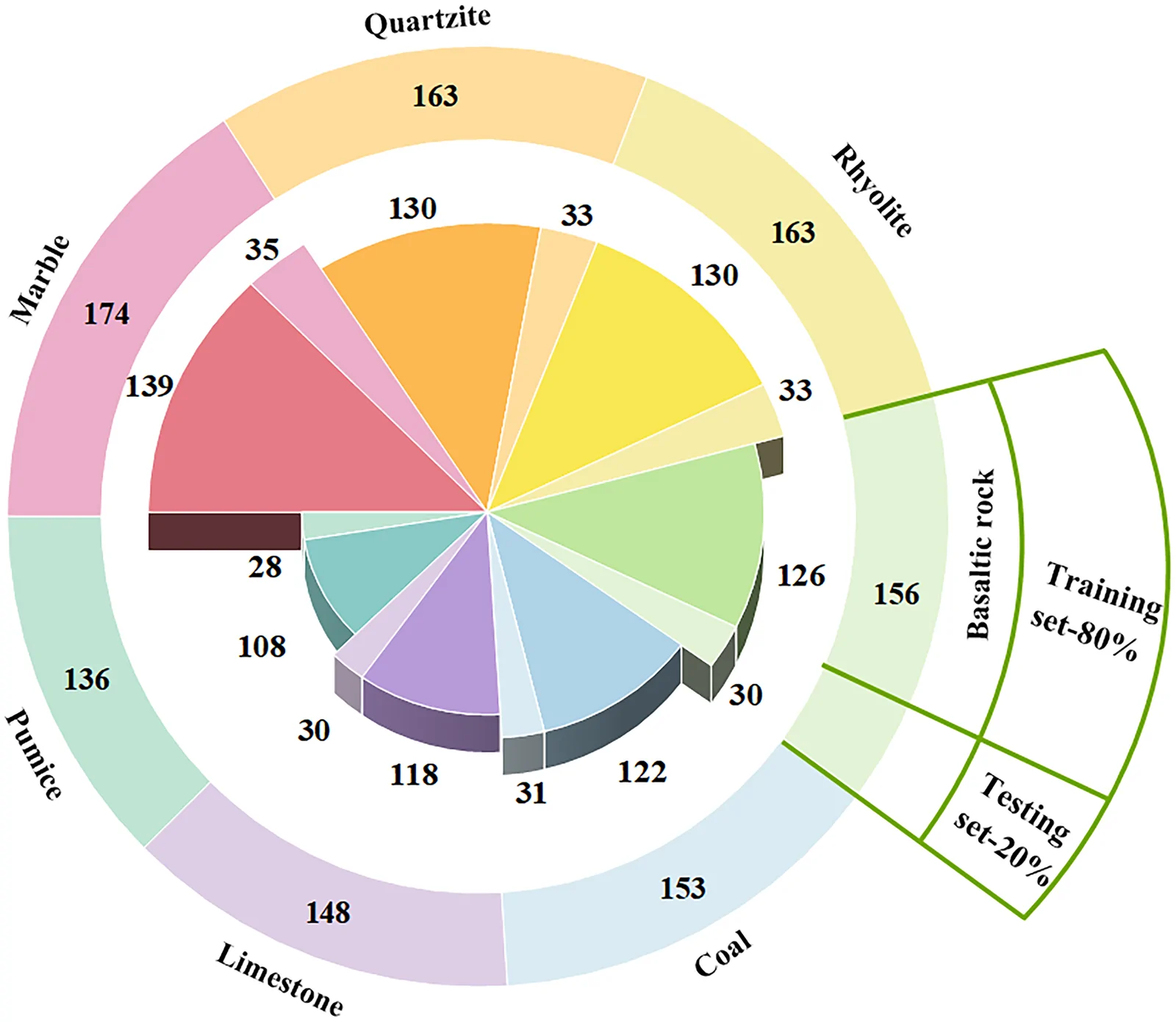
Distribution of specific numbers of data sets.

To achieve fast training convergence and improve model generalization, all images were first subjected to identical preprocessing: grayscale conversion, histogram equalization, and resizing to a standardized resolution of 224 × 224 pixels, followed by conversion to a tensor format and normalization. To further augment the training data and mitigate overfitting, random flipping and cropping were applied during training using the PyTorch toolbox. The dataset was constructed by merging rock image collections from two publicly available sources (Kaggle and Roboflow) to increase sample diversity. Although the original images vary in resolution, all images were uniformly resized to 224 × 224 pixels, ensuring consistent input dimensions. Camera specifications (sensor size, focal length) and illumination conditions (natural daylight vs. controlled indoor lighting) are not explicitly documented for these open-source datasets, which may introduce potential domain shift. To mitigate this, grayscale conversion and histogram equalization were applied to reduce the impact of illumination and color variations, and random flipping and cropping were used to improve robustness. Furthermore, the stratified 5-fold cross-validation scheme (detailed below) ensures that each fold contains a representative distribution of both sources, reducing evaluation bias. A more systematic domain adaptation analysis will be explored in future work.

After dataset construction and cleaning, we first performed repeated 8:2 random train-test splits to preliminarily evaluate model performance and verify the rationality of the cleaned dataset. To further eliminate the randomness introduced by a single partition and rigorously verify the stability of model performance, we subsequently adopted a 5-fold cross-validation strategy on the lithology dataset. All performance evaluations are based on 5-fold stratified cross-validation to ensure the reliability of the results. Specifically, the entire dataset is randomly shuffled and partitioned into five mutually exclusive folds using stratified sampling, which preserves the proportion of each lithology category in every fold and minimizes the risk of data leakage from shared image sources. In each validation round, four folds are used for model training and the remaining fold is used for testing. This process is repeated five times until each fold has been used as the test set exactly once. The final reported precision, recall, accuracy, and F_1_-score are the arithmetic mean of the five test results, and the corresponding standard deviation is also provided to reflect the stability of model performance.

As shown in Table 1, the average accuracy of the 5-fold cross-validation is 81.56% ± 2.11%, with minor fluctuations across all folds. The corresponding average recall, precision, and F_1_-score are 81.90% ± 2.08%, 82.02% ± 1.97%, and 81.75% ± 2.06%, respectively. The low standard deviation confirms that the model maintains stable performance across different splits, with no obvious overfitting or overestimation issues. This validates the rationality of the cleaned dataset partitioning and demonstrates that the proposed ResNet18-CBAM model has good robustness in lithology classification, laying a solid foundation for the reliability of subsequent model comparison and generalization experiments.

**Table 1:** 5-Fold cross-validation results.

Fold	Accuracy	Recall	Precision	F_1_-score
1	82.86%	83.31%	82.92%	82.81%
2	81.71%	81.68%	82.40%	81.81%
3	78.29%	78.80%	78.91%	78.57%
4	80.46%	80.83%	81.07%	80.80%
5	84.48%	84.88%	84.81%	84.77%
Average ± Std. Dev.	81.56% ± 2.11%	81.90% ± 2.08%	82.02% ± 1.97%	81.75% ± 2.06%

### 4.3 Comparative analysis of multiple attention mechanisms

Selecting an appropriate attention mechanism is important for training the model. The attention mechanism helps the model focus on key information and on the region where the target object is located in an image. This reduces interference from irrelevant details and enables the model to extract object-related features more accurately. In addition, an appropriate attention mechanism can reduce computation time, improve model efficiency, accelerate convergence, and enhance the model’s ability to generalize. This study selects five attention mechanisms, namely CBAM, channel, ECA, SE, and spatial, and incorporates them into the ResNet18 model for training. The accuracy, loss value, recall, precision, and F_1_-score are used to compare the effects of these five attention mechanisms on model precision. The test results are presented in Figs 9–11.

**Fig 9:**
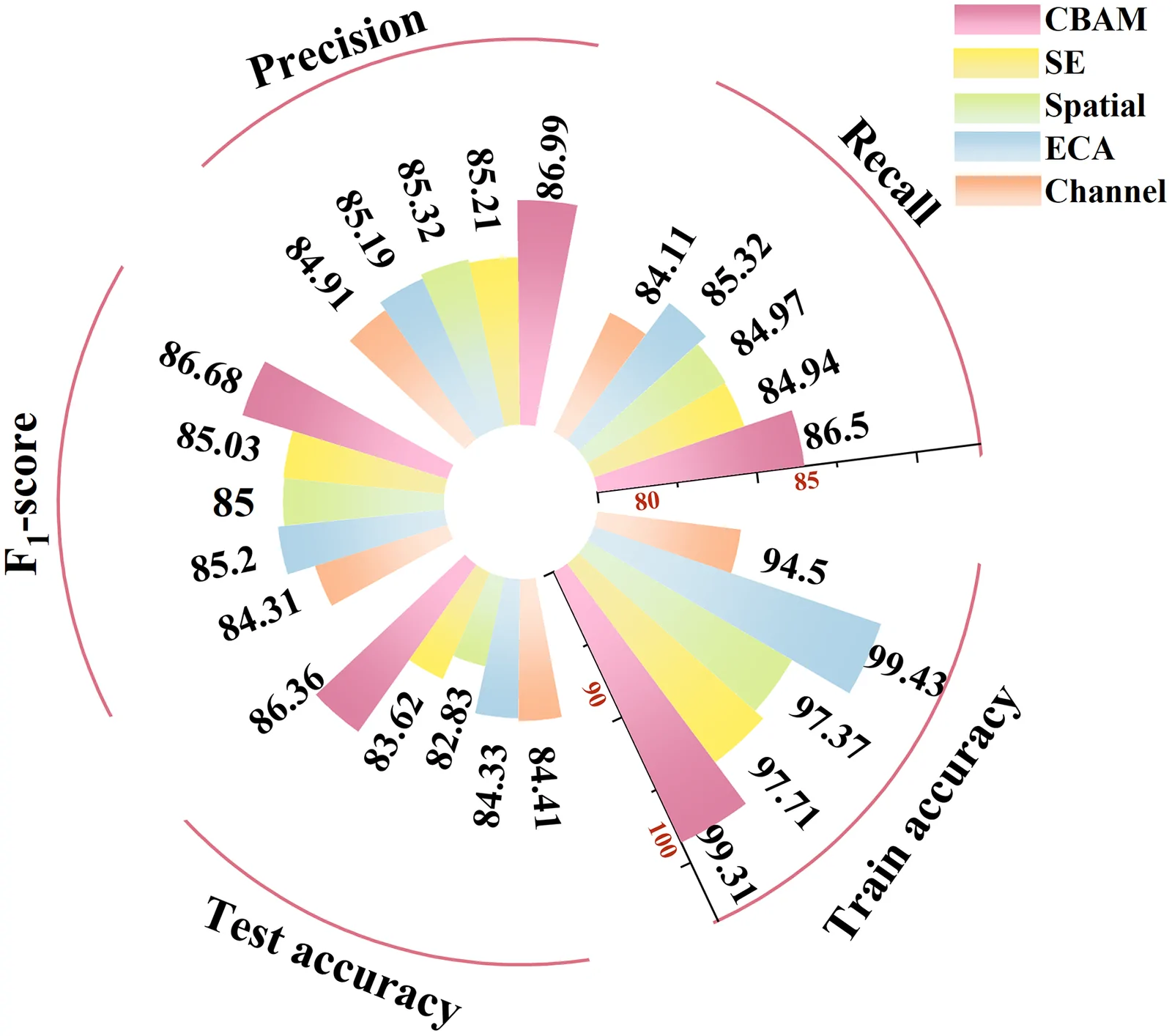
Performance comparison of five attention mechanisms (CBAM, SE, Spatial, ECA, Channel) across Precision, Recall, F_1_-score, training accuracy and test accuracy.

**Fig 10:**
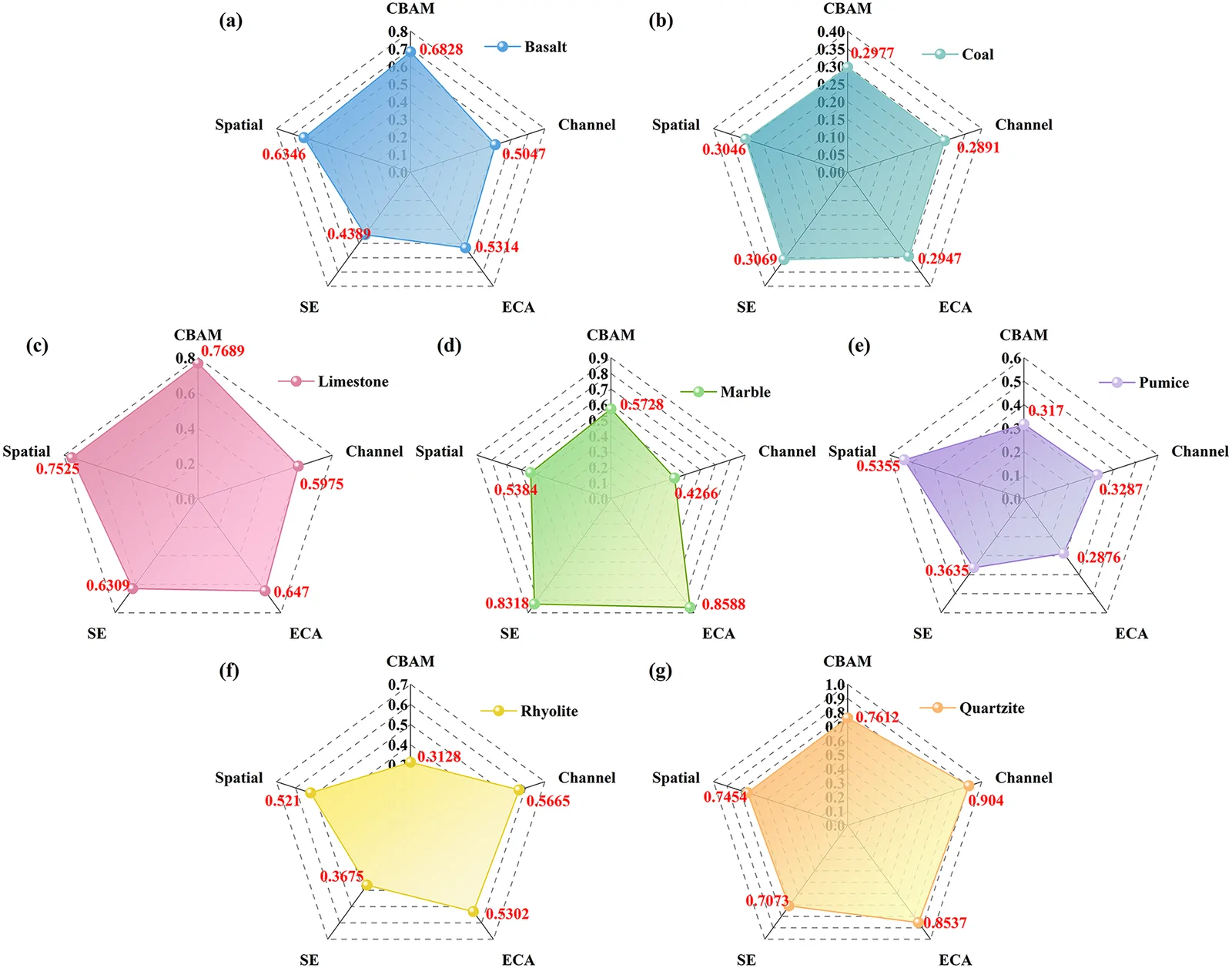
The impact of five attention mechanisms on the training of seven rock types was analyzed via loss rates. (a) Loss rate of basalt. (b) Loss rate of coal. (c) Loss rate of limestone. (d) Loss rate of calcitic marble. (e) Loss rate of rhyolitic pumice. (f) Loss rate of quartzite. (g) Loss rate of rhyolite.

**Fig 11:**
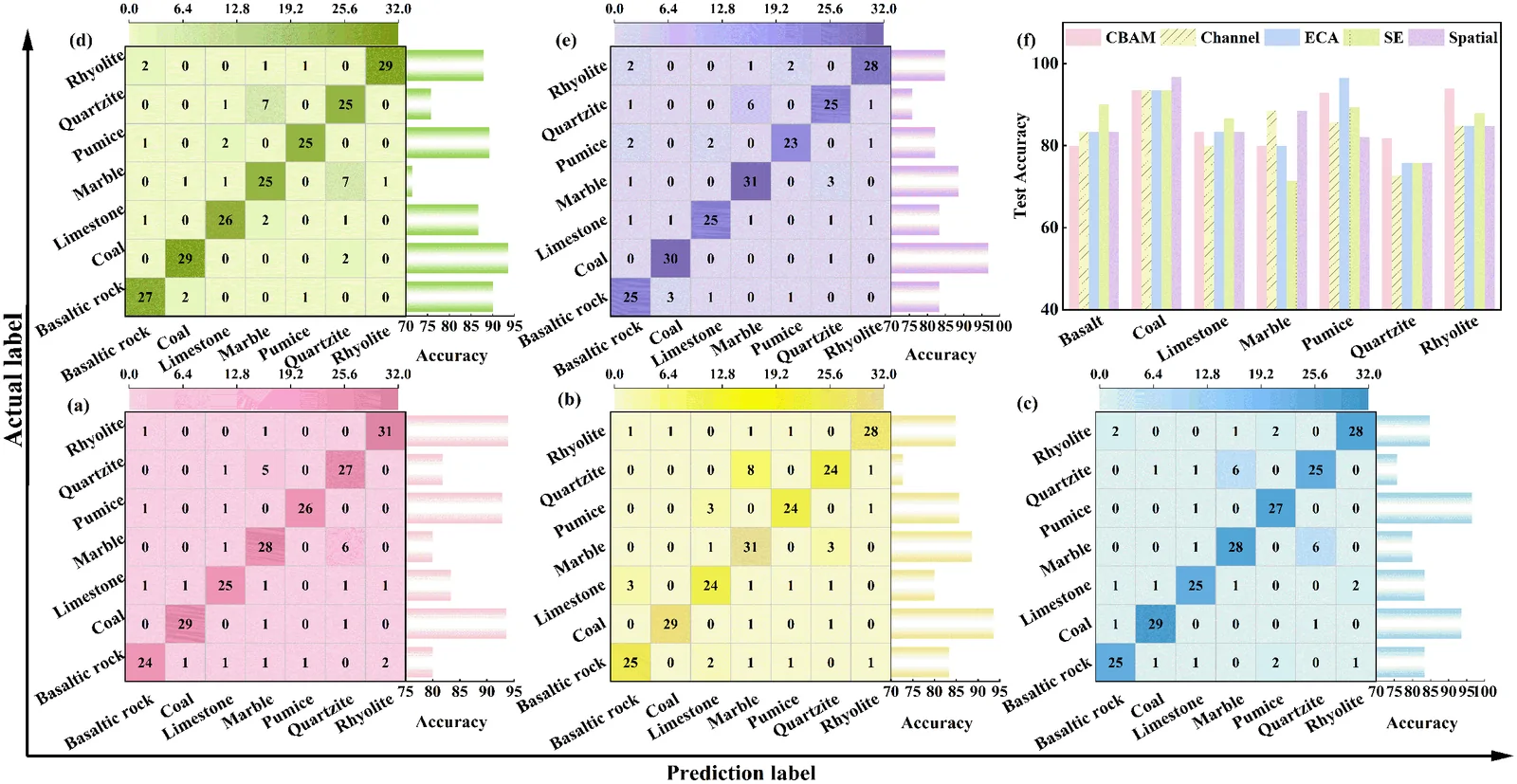
The influence of five attention mechanisms on the model’s rock identification performance is analyzed using confusion matrix plots. (a) Confusion matrix of the CBAM attention mechanism. (b) Confusion matrix of the channel attention mechanism. (c) Confusion matrix of the ECA attention mechanism. (d) Confusion matrix of the SE attention mechanism. (e) Confusion matrix of the spatial attention mechanism. (f) Comparison of recognition accuracy among the five attention mechanisms for seven rock types.

The model is trained for 100 epochs, and the accuracy, recall, precision, and F_1_-score of the five different attention mechanisms eventually stabilize. The experimental results are calculated at the fixed final epoch after model convergence, and the average values over multiple runs are obtained. As shown in Fig 9, the CBAM attention mechanism achieves a test accuracy of 86.36% and a training accuracy of 99.31%, followed by the SE attention mechanism with a test accuracy of 83.62% and a training accuracy of 97.71%; the spatial attention mechanism with a test accuracy of 82.83% and a training accuracy of 97.37%; the ECA attention mechanism with a test accuracy of 84.33% and the highest training accuracy of 99.43%; and the channel attention mechanism with a test accuracy of 84.41% and a training accuracy of 94.50%. In terms of recall, the CBAM attention mechanism achieves the highest recall of 86.50%, followed by the ECA attention mechanism at 85.32%, the spatial attention mechanism at 84.97%, the SE attention mechanism at 84.94%, and the channel attention mechanism at 84.11%. In terms of precision, the CBAM attention mechanism has the highest precision of 86.99%, followed by the spatial attention mechanism at 85.32%, the SE attention mechanism at 85.21%, the ECA attention mechanism at 85.19%, and the channel attention mechanism at 84.91%. In terms of F_1_-score, the CBAM attention mechanism achieves the highest F_1_-score of 86.68%, followed by the ECA attention mechanism at 85.20%, the SE attention mechanism at 85.03%, the spatial attention mechanism at 85.00%, and the channel attention mechanism at 84.31%. Overall, the CBAM attention mechanism outperforms the other attention mechanisms in terms of test accuracy, recall, precision, and F_1_-score, and it exhibits competitive training accuracy that is very close to the highest value achieved by the ECA attention mechanism.

The observed gap between training and test accuracy suggests some degree of overfitting, which is a common challenge when training deep models on small-scale datasets. In this study, we employed basic data augmentation (random rotation and horizontal flipping) and early stopping to partially mitigate this issue. Systematic regularization techniques such as dropout, weight decay, or advanced augmentation strategies (e.g., MixUp, CutMix) were not extensively explored in the current benchmark-focused experiment. We recognize that incorporating these techniques could further improve generalization, and we plan to evaluate them in future work.

Fig 10 shows the training loss rates of the five attention mechanisms for each of the seven rock types.

For basalt training, the loss rates of the five attention mechanisms are as follows: CBAM (0.6828), Channel (0.5047), ECA (0.5314), SE (0.4389), and Spatial (0.6346). SE attention achieves the lowest and most stable loss rate, showing the best adaptability for basalt training, while CBAM presents the highest loss value among the five modules.

For coal training, the loss rates are all relatively low across the five mechanisms: CBAM (0.2977), Channel (0.2891), ECA (0.2947), SE (0.3069), and Spatial (0.3046). Channel attention achieves the minimum loss, and all five attention mechanisms perform well for coal, with only slight differences in convergence performance.

For limestone training, the loss rates are CBAM (0.7689), Channel (0.5975), ECA (0.6470), SE (0.6309), and Spatial (0.7525). Channel attention outperforms the others for limestone identification with the lowest training loss, while ECA ranks third among the five mechanisms.

For calcitic marble training, the loss rates are CBAM (0.5728), Channel (0.4266), ECA (0.8588), SE (0.8318), and Spatial (0.5384). Channel attention achieves the lowest loss rate, showing the best adaptability for calcitic marble training. In contrast, ECA and SE present the two highest loss values with relatively slow convergence.

For rhyolitic pumice training, the loss rates are CBAM (0.3170), Channel (0.3287), ECA (0.2876), SE (0.3635), and Spatial (0.5355). All five attention mechanisms show relatively low and controllable loss rates for rhyolitic pumice training, with ECA and CBAM exhibiting the best performance.

For rhyolite training, the loss rates are CBAM (0.3128), Channel (0.5665), ECA (0.5302), SE (0.3675), and Spatial (0.5210). CBAM and SE attention show the lowest loss rates for rhyolite training.

For quartzite training, the loss rates are CBAM (0.7612), Channel (0.9040), ECA (0.8537), SE (0.7073), and Spatial (0.7454). SE and spatial attention achieve the lowest loss rates, showing better adaptability for quartzite identification.

Based on the above analysis, the training loss rates vary significantly across different rock types. Channel attention performs stably well on most lithologies, especially on calcitic marble and limestone with obvious directional texture features. SE attention shows clear advantages on lithologies with prominent local spatial features, achieving the lowest loss on both basalt (0.4389) and quartzite (0.7073). In contrast, pure spatial attention exhibits relatively high training loss on basalt (0.6346, the second highest among the five mechanisms) despite achieving competitive performance on quartzite (0.7454). As a dual-branch attention module, CBAM shows moderate loss performance on a single lithology but achieves the best comprehensive classification accuracy across all seven categories, which benefits from its balanced modeling ability for both channel and spatial features. These results indicate that different attention mechanisms exhibit varying degrees of adaptability to different rock types, and no single mechanism performs optimally across all categories.

The performance differences of attention mechanisms across lithologies are closely related to the visual feature attributes of rock images. CBAM integrates both channel and spatial attention branches, which can simultaneously capture channel-wise feature responses and spatial position information. For the whole dataset with diverse texture types, this dual-dimensional feature enhancement enables CBAM to adapt to different lithological feature patterns, thus achieving the best overall performance.

Single-branch attention mechanisms show obvious lithology selectivity. For lithologies with uniform texture and prominent global features (such as marble with strip texture), channel attention can effectively weight key feature channels, thus achieving excellent performance. For lithologies with complex local details and significant spatial distribution differences (such as basalt with pore distribution), SE attention can better focus on local discriminative regions and achieves the lowest training loss, whereas pure spatial attention shows limited improvement on basalt with relatively high loss despite its theoretical suitability for spatial feature enhancement. However, single-branch attention cannot simultaneously account for both channel and spatial characteristics, so its performance fluctuates greatly across different lithologies.

As shown in Fig 11, the influence of the five types of attention mechanisms on model lithology identification is analyzed using confusion matrix plots. The performance of the attention mechanisms is visualized through confusion matrices, which reflect error patterns via diagonal occupancy for correct predictions and non-diagonal concentrations for misclassifications. The CBAM attention mechanism demonstrates competitive performance across most lithologies: it achieves 80.00% for basalt, 93.55% for coal, 83.33% for limestone, 80.00% for calcitic marble, 92.86% for rhyolitic pumice, 81.82% for quartzite, and 93.94% for rhyolite, yielding a mean per-class accuracy of 86.50%. The ECA attention mechanism shows an average accuracy of 85.32%, with balanced results across categories: it reaches 93.55% for coal, 96.43% for rhyolitic pumice, and its limestone accuracy matches that of CBAM at 83.33%. The SE attention mechanism exhibits stable performance with an average accuracy of 84.94%. It achieves the highest basalt accuracy at 90.00%, 93.55% for coal, 86.67% for limestone, 89.29% for rhyolitic pumice, 75.76% for quartzite, and 87.88% for rhyolite, though its calcitic marble identification is relatively low at 71.43%. The spatial attention mechanism shows an average accuracy of 84.96%. It performs best on coal at 96.77%, with 83.33% for basalt and limestone, 88.57% for calcitic marble, 82.14% for rhyolitic pumice, 75.76% for quartzite, and 84.85% for rhyolite. Notably, while CBAM achieves strong overall performance, its accuracy on basalt and calcitic marble (80.00%) is slightly lower than that of other mechanisms such as SE (90.00% for basalt) and Channel (88.57% for calcitic marble). However, CBAM maintains robust performance on rhyolitic pumice (92.86%) and rhyolite (93.94%), and its combined channel-spatial attention effectively balances feature extraction across different lithologies. Notably, misclassifications between basalt and calcitic marble arise from their visual similarity under grayscale preprocessing: both exhibit medium-gray tonal ranges, and the directional strip texture of marble may not be sufficiently distinct in low-resolution or poorly illuminated samples. Similarly, quartzite and rhyolite share comparable granular appearances, leading to occasional confusion. These error patterns highlight the need for higher-resolution imaging or complementary spectral features in future work.

Therefore, in comparison with the other attention mechanisms, the CBAM attention mechanism exhibits superior efficacy in mitigating overfitting risks and accelerating model convergence. From the perspective of lithological image characteristics, the core discriminative information for lithology identification essentially comes from spatially distributed geological features, including grain boundaries, bedding structures, mineral aggregate morphology, and surface weathering textures. These diagnostic features are manifested as local spatial differences in texture and grayscale distribution, rather than global channel-level statistical differences.

Specifically, the grayscale preprocessing adopted in this study weakens color interference and further highlights the spatial distribution characteristics of textural and structural features. The spatial attention component of CBAM can adaptively assign higher weights to local regions with prominent geological structural features, enabling targeted enhancement of key discriminative areas. In contrast, pure channel attention mechanisms such as SE and ECA only perform global weight adjustment along the channel dimension and cannot locate or strengthen the local spatial texture regions that determine lithology classification. This inherent limitation reduces their ability to capture fine-grained lithological differences and results in weaker overall adaptability to complex rock images.

Collectively, the spatial-channel dual attention mechanism of CBAM matches the spatial essence of lithological geological features more closely than single-channel attention, which explains its superior comprehensive performance in lithology identification tasks.

To further interpret the model’s decision-making process and verify that the attention mechanism focuses on geologically relevant features rather than background noise, we utilized Grad-CAM to visualize the feature heatmaps of the trained ResNet18-CBAM model across the seven rock categories (Fig 12). The visualization results show that the high-activation regions (red/yellow areas) predominantly overlap with the core textures and structural features of the rocks, such as the bedding structure of limestone, the banded texture of calcitic marble, and the compact massive structure of basalt, while largely ignoring background distractions. This confirms that the proposed ResNet18-CBAM model effectively captures geologically meaningful patterns for lithology identification.

**Fig 12:**
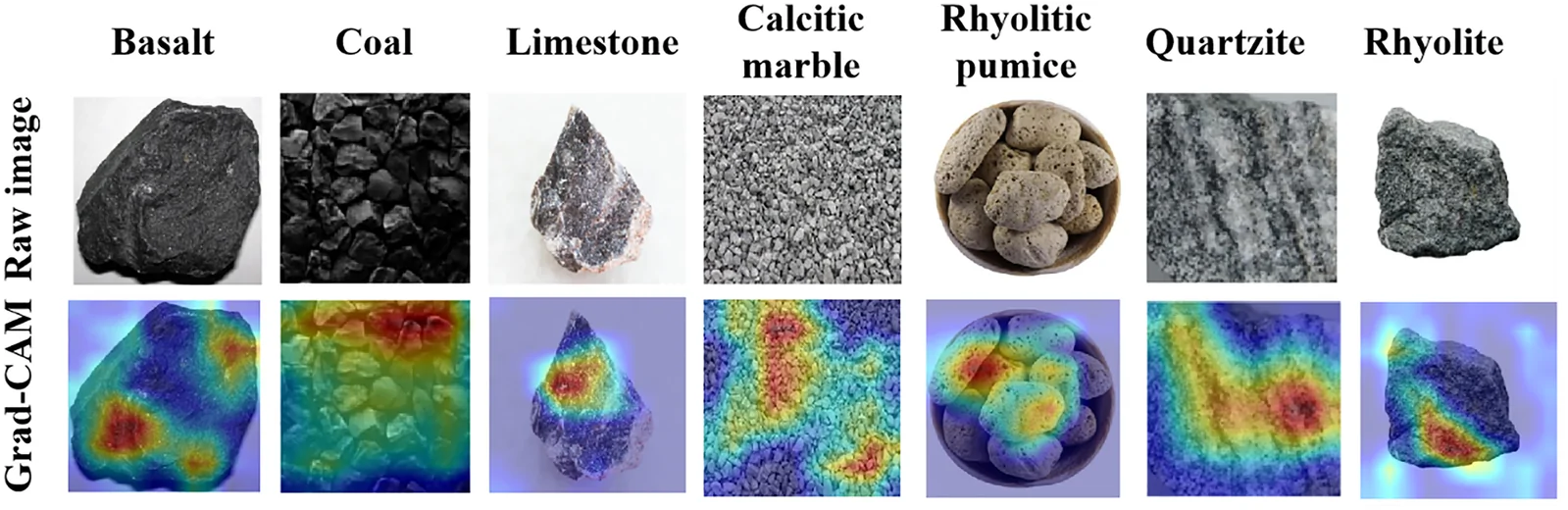
Grad-CAM heatmap visualization for seven rock categories.

## 5. Hyperparameter sensitivity analysis

### 5.1 Learning rate and batch size selection

This study uses the Adam optimizer, a widely used optimization algorithm for deep learning that can adaptively adjust the learning rate of each parameter. It is stable, efficient, and easy to implement. In model training, selecting a reasonable learning rate is crucial because a low learning rate causes the model to converge very slowly, while a high learning rate prevents the model from converging, resulting in the loss function not obtaining an optimal solution. For training and learning the ResNet18 model on a small dataset, the learning rate is set to 0.0001, 0.0003, and 0.0005. Although batch size has a relatively small impact on model test accuracy and training loss compared with the learning rate, it is a key hyperparameter in model training and is critical for improving model stability and reducing fluctuations. Based on this, this study selects batch sizes of 16, 32, and 64 and learning rates of 0.0001, 0.0003, and 0.0005. Each batch size is tested against each learning rate to compare their impact on model accuracy and loss. The specific test results are shown in Fig 13.

**Fig 13:**
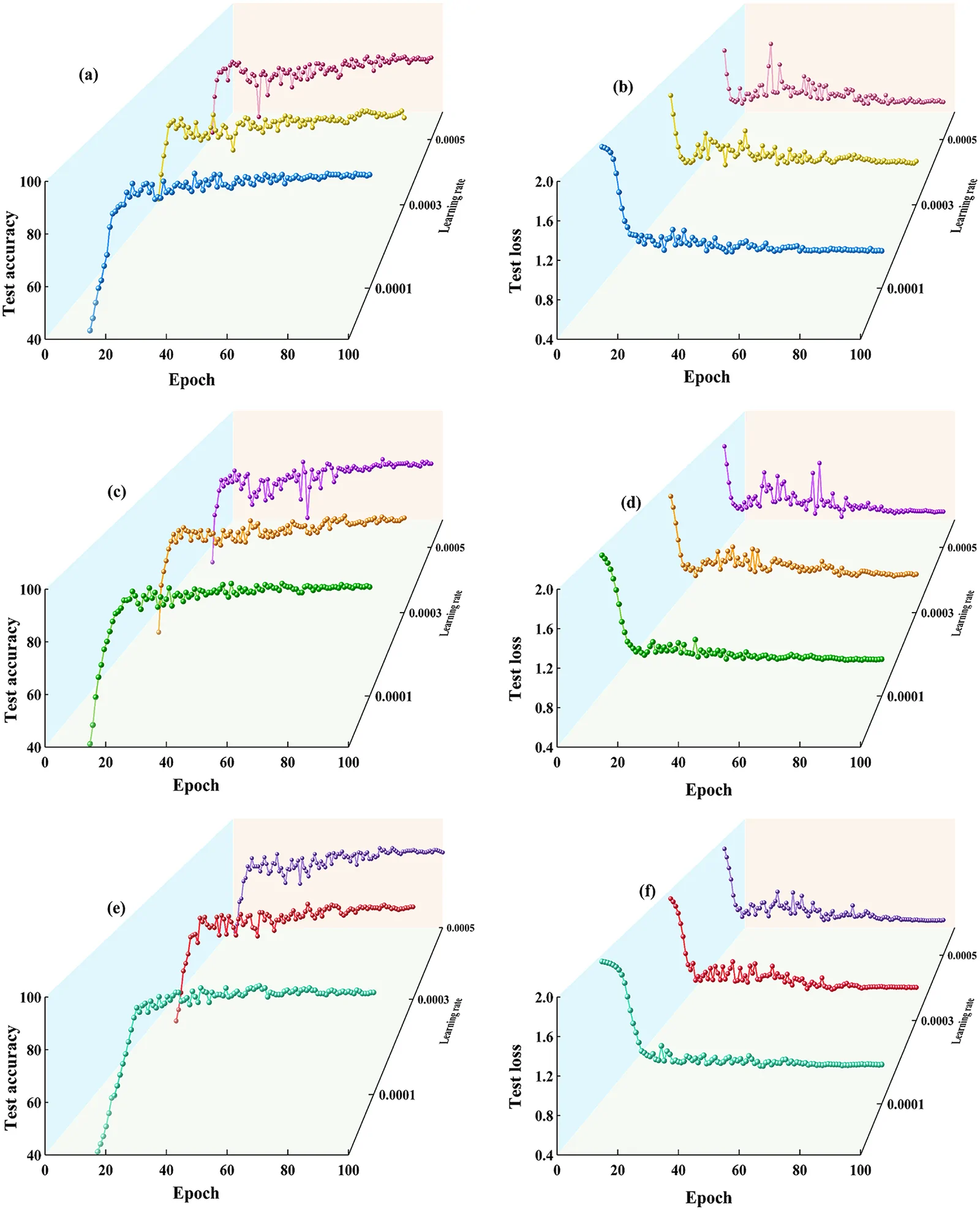
Model performance metrics under different combinations of batch sizes and learning rates. (a) Accuracy corresponding to three learning rates when the batch size is 16. (b) Loss values corresponding to three learning rates when the batch size is 16. (c) Accuracy corresponding to three learning rates when the batch size is 32. (d) Loss values corresponding to three learning rates when the batch size is 32. (e) Accuracy corresponding to three learning rates when the batch size is 64. (f) Loss values corresponding to three learning rates when the batch size is 64.

In this round, the training batch size is set to 16, and training structures with learning rates of 0.0001, 0.0003, and 0.0005 are compared. When the learning rate is set to 0.0001, the model achieves 86.36% accuracy, which is the highest among the three learning rates, and the loss value reduces to 0.8 after 71 epochs before stabilizing. When the learning rate is set to 0.0003, the model achieves an 83.64% accuracy, fluctuating greatly in the early stage, and the loss value reduces to 0.93 after 84 epochs before stabilizing. When the learning rate is set to 0.0005, the model’s accuracy reaches 84.09%, with the accuracy fluctuating greatly and stabilizing after 78 epochs, and the loss value decreasing to 0.93 and stabilizing after 80 epochs.

In this round, the training batch size is set to 32, and training structures with learning rates of 0.0001, 0.0003, and 0.0005 are compared. When the learning rate is set to 0.0001, the model’s accuracy reaches 85.45%. However, the accuracy fluctuates more over 40 epochs, and the loss value decreases to 0.8 after 76 epochs, after which it stabilizes. When the learning rate is set to 0.0003, the model’s accuracy reaches 84.55%, with the accuracy fluctuating less after 71 epochs and the loss value decreasing to 0.88 after 85 epochs; the fluctuation becomes smaller and the model gradually stabilizes. When the learning rate is set to 0.0005, the model’s accuracy decreases to 84.55%. After 79 epochs, the accuracy fluctuates less and stabilizes, and the loss value decreases to 0.88 after 73 epochs and gradually stabilizes.

In this round, the training batch size was set to 64, and models with learning rates of 0.0001, 0.0003, and 0.0005 were compared. At a learning rate of 0.0001, the model achieved 83.18% accuracy, and the loss value stabilized at 0.83 after 69 epochs. When the learning rate was 0.0003, the model also reached 85.45% accuracy, with reduced fluctuations after 71 epochs and a stabilized loss value of 0.81 at the 77th epoch. Increasing the learning rate to 0.0005 led to an accuracy of 82.73%, where the accuracy stabilized with minimal fluctuations after 77 epochs, and the loss value gradually decreased to 0.89 at the 69th epoch.

In summary, a comparative analysis of nine sets of experimental data on hyperparameter sensitivity, combined with an assessment of model training accuracy and training loss convergence, indicates that the ResNet18 model fused with the CBAM attention mechanism achieves optimal performance on this lithology dataset when the batch size is set to 16 and the learning rate to 0.0001.

## 6. Discussion

### 6.1 Model Comparison Analysis

To further verify the advancement and effectiveness of the proposed ResNet18-CBAM model, we conducted comprehensive comparative experiments with the baseline ResNet18, mainstream classical CNN models, and existing lithology classification models, with all experiments carried out under the same dataset and experimental settings. As shown in Table 2, the ResNet18-CBAM model achieves the highest test accuracy of 86.36%, outperforming the original ResNet18 (84.55%) by 1.81%, which confirms that the CBAM attention mechanism effectively enhances the feature extraction ability of the baseline model by focusing on the key channel and spatial information of rock images.

**Table 2:** Model comparison experiments.

Model	Test accuracy	Test precision	Test recall	Test F_1_-score	Test loss
ResNet18-CBAM	86.36%	86.99%	86.5%	86.68%	0.8354
ResNet18	84.55%	84.79%	84.76%	84.71%	0.8331
VGG16	82.73%	82.23%	82.58%	82.29%	0.8909
MobileNetv2	81.36%	81.86%	81.76%	81.61%	0.8533
Inceptionv3	83.64%	82.28%	81.80%	81.83%	0.8843
EfficientNetV3	84.55%	84.99%	84.94%	84.62%	0.8536
DenseNet121	82.73%	83.34%	83.05%	83.08%	0.8489
GoogleNet	83.64%	84.02%	83.86%	83.82%	0.8740
SqueezeNet	80.45%	80.52%	80.63%	80.50%	0.9535
ShuffleNetV2	84.55%	84.62%	84.76%	84.52%	0.8480
RSHFNet	84.09%	84.56%	84.29%	84.31%	0.8094
FSHNet	84.09%	84.76%	84.39%	83.94%	0.8776

In comparison with mainstream CNN models including VGG16 [42], MobileNetV2 [43], InceptionV3 [44], EfficientNetV3 [45], DenseNet121 [46], GoogleNet [47], SqueezeNet [48], and ShuffleNetV2 [49], the proposed model still ranks first in all core evaluation metrics (precision, recall, F_1_-score, and test accuracy). Among them, lightweight models such as MobileNetV2 and SqueezeNet have relatively low accuracy due to insufficient feature extraction depth, while complex models like VGG16 and InceptionV3 suffer from overfitting on the lithology dataset, leading to limited performance improvement. In addition, compared with the existing specialized lithology classification models RSHFNet [50] and FSHNet [51] (both 84.09%), our ResNet18-CBAM model also shows obvious advantages, which proves that the lightweight improvement strategy integrating CBAM is more suitable for rock image identification tasks and has strong competitiveness in the field of lithology classification.

It is acknowledged that the current dataset size may impose limitations on the model’s generalization capability to the broader diversity of geological conditions. The collection and labeling of high-quality rock images are inherently resource-intensive, creating significant challenges for assembling large-scale datasets in lithological studies. In line with recent findings regarding deep learning applications in resource-constrained scenarios (Shahinfar et al. [52]), this study serves as a baseline investigation to demonstrate the feasibility of automated rock classification under practical limitations. The current work focuses on establishing a robust methodological framework rather than achieving state-of-the-art performance on an exhaustive dataset. Future research will prioritize the curation of larger, standardized datasets encompassing a wider array of depositional environments and weathering states to further validate and enhance the model’s transferability and robustness.

### 6.2 Effect after dataset expansion

To alleviate the impact of small sample size on model generalization and verify the stability of the proposed method, we expanded and cleaned the original dataset, and the detailed sample distribution is presented in Table 3. The expanded dataset contains 3,199 rock images covering seven categories, with a balanced distribution of training set (2,559 images) and test set (640 images), which effectively avoids the overfitting risk caused by insufficient data and sample imbalance.

**Table 3:** Sample distribution of the expanded dataset.

Rock category	Training set	Test set	Total
Basalt	360	90	450
Coal	372	93	465
Limestone	350	88	438
Calcitic marble	365	91	456
Rhyolitic pumice	358	89	447
Quartzite	380	95	475
Rhyolite	374	94	468
Total	2559	640	3199

The 5-fold cross-validation results on the expanded dataset (Table 4) show that the model achieves an average accuracy of 95.58% ± 0.66%, with a small standard deviation and stable performance across each fold, indicating that the model has strong robustness and generalization ability. The final test results (Table 5) illustrate that the ResNet18-CBAM model reaches a test accuracy of 90.78% on the expanded dataset, which is significantly improved compared with the result on the original cleaned dataset. This fully demonstrates that reasonable dataset expansion and standardized preprocessing can effectively enhance the feature learning effect of the model, and the proposed improved ResNet18 model can still maintain excellent identification performance under large-scale data, further validating the reliability and practical application value of the method in actual geological engineering scenarios.

**Table 4:** 5-Fold cross-validation results.

Fold	Accuracy	Recall	Precision	F_1_-score
1	95.31%	95.37%	95.41%	95.34%
2	96.68%	96.68%	96.81%	96.72%
3	95.90%	95.91%	96.04%	95.95%
4	94.73%	94.77%	94.82%	94.72%
5	95.30%	95.35%	95.33%	95.33%
Average ± Std. Dev.	95.58% ± 0.66%	95.61% ± 0.72%	95.68% ± 0.77%	95.61% ± 0.76%

**Table 5:** Model training results.

Model	Test accuracy	Test precision	Test recall	Test F_1_-score	Test loss
ResNet18-CBAM	90.78%	90.23%	90.15%	90.13%	0.7140

### 6.3 Limitations and future work

Environmental adaptability and surface texture variability. In real-world geological surveys, rock surface appearance can be significantly affected by environmental factors such as moisture content, surface weathering, and ambient lighting [53]. The visual appearance of rocks is highly susceptible to microclimatic factors, surface moisture, and weathering, which alter texture and structural clarity. For instance, increased moisture darkens surfaces and obscures grain boundaries, while weathering induces roughness that affects feature extraction. Recent environmental data modeling studies [54] highlight how climatic variables drive surface feature changes, emphasizing the need to integrate such metadata into geological interpretations. Although our controlled dataset does not fully capture these dynamics, we recognize that evaluating model robustness under such complex physical conditions is essential for practical deployment. We acknowledge their impact and will incorporate environmental parameters in future work to strengthen the geological validity of our approach. Future work will also explore domain adaptation techniques to enhance the model’s resilience to field conditions.

Class imbalance consideration. The dataset exhibits slight class imbalance, which may bias overall accuracy toward majority categories and increase misclassification risk for visually similar lithologies (e.g., quartzite vs. rhyolite). In this study, standard cross-entropy loss was used. Future work will explore class-weighted cross-entropy or focal loss to better handle class imbalance and reduce confusion among similar rock types.

Data scale, rare lithologies, and edge deployment. The current dataset primarily covers typical lithologies in karst areas, and the generalization ability for rare rock types still needs further verification. Subsequent research will expand the sample coverage of special lithologies and introduce few-shot learning strategies to improve the model’s adaptability to scarce samples. Additionally, for real-world deployment, the model must operate under edge device constraints (e.g., limited memory and computational power). Model quantization (e.g., 8-bit integer quantization) can significantly reduce model size and inference latency with minimal accuracy drop, enabling real-time lithology identification on portable field devices. Future work will evaluate the trade-offs between quantization precision and inference speed under variable outdoor lighting conditions.

## 7. Conclusions

This study systematically benchmarks five lightweight attention mechanisms integrated into the ResNet18 backbone for lithology image classification and identifies CBAM as the optimal module on the tested public rock image dataset.

In terms of theoretical implications, the performance gap between spatial-channel joint attention and pure channel attention is closely associated with the inherent properties of lithological images. Core discriminative features of rock samples, such as grain boundaries, bedding structures, weathering textures, and mineral distribution, are mainly presented as spatially distributed local visual patterns. This feature makes attention mechanisms with spatial weighting capability more adaptable to lithology identification tasks than channel-only schemes, which provides a useful reference for feature-driven model design in geological image recognition scenarios.

For practical engineering applications, the proposed lightweight CNN-attention framework achieves a reasonable balance between recognition accuracy and computational cost. With negligible parameter increment and limited computational overhead, the model has the potential to be deployed on portable edge devices commonly used in geological field surveys. Combined with the grayscale conversion and histogram equalization preprocessing pipeline validated in this study, the method shows acceptable robustness to illumination variations and may provide auxiliary reference for lithology judgment in field geological mapping, engineering survey, and mineral exploration scenarios.

Several limitations of this study should be noted. First, the scale of the adopted public dataset is relatively limited, and the generalization performance of the model under complex field conditions (such as variable weathering, surface moisture, and natural illumination) remains to be further verified. Second, this work only conducts comparative research based on the CNN backbone and lacks cross-architecture comparison with attention-native lightweight models. Third, the current model relies only on visual features for identification, without integrating multi-source geological attribute information.

Accordingly, future research will focus on the following aspects: (1) explore synthetic sample generation and unsupervised domain adaptation methods to improve the model’s adaptability to unseen field conditions; (2) investigate learning rate scheduling strategies such as cosine annealing to stabilize training convergence and carry out cross-architecture benchmark comparisons with lightweight vision transformers; (3) construct a multi-source fusion lithology identification model combining visual features with physical and chemical geological parameters; (4) validate the performance of the quantized model on actual portable edge devices to further evaluate its practical application value in field surveys.
